# Supplementary material for: The Genus Kalanchoe (Crassulaceae) in Ecuador: From Gardens to the Wild
Source: Plants (Basel). 2022 Jun 30;11(13):1746. doi: 10.3390/plants11131746 (PMC9269312; doi:10.3390/plants11131746)
Supplement: Supplementary file 1 [file plants-11-01746-s001.zip › plants-1779913-supplementary/Text_S1.docx]

**Text S1.** Nomenclatural considerations in the genus *Kalanchoe* and the studied species.

***Kalanchoe*** Adans., *Fam. Pl*. 2: 248 (1763).

Valid synonyms with designated type:

***Vereia*** Andrews, *Bot. Repos*. 1: t. 21 (1798), type: *Vereia crenata* Andrews [orthographic variant: ***Verea*** Willd., *Sp. Pl*. ed 4, 2(1): 471 (1799)].

***Bryophyllum*** Salisb., *Parad. Lond*.: pl. 3 (1805), type: *Bryophyllum calycinum* Salisb.

***Geaya*** Costantin & Poiss., *Compt. Rend. Hebd. Séances Acad. Sci.* 147: 636 (1908), type: *Geaya purpurea* Costantin & Poiss.

Valid synonyms without designated type:

***Kitchingia*** Baker, *J. Linn. Soc., Bot*. 18: 268 (1881).

Orthographic variants:

***Calanchoe*** Pers., *Syn. Pl*. 1: 445 (1805).

***Kalenchoe*** Haw., *Syn. Pl. Succ*.: 109 (1812).

[Based in: Tropicos (<https://www.tropicos.org>), African Plant Database (version 4.0.0, <https://africanplantdatabase.ch>), and Smith, G.F.; Figueiredo, E. & van Wyk, A.E. 2019. *Kalanchoe (Crassulaceae) in southern Africa. Classification, biology and cultivation*. Academic Press. Elsevier Inc. 328 p.]

*Kalanchoe* Adans. is now widely accepted to designate the genus. However, Berger [1] recognized that the species included in *Kalanchoe* (sensu lato) actually belonged to two genera, whether to *Kalanchoe* (sensu stricto) or *Bryophyllum*. This latter idea was followed in the majority of recent floristic treatments (e.g. [2,3]).

The modern synoptic revision of *Kalanchoe* by Descoings [4] provides two sections within the genus, despite the author admited that some species cannot be unambiguously placed in one of these two sections:

1. Sect. *Kalanchoe*: leaves and inflorescences never with bulbils, among another characters.

2. Sect. *Bryophyllum* (Salisbury) Boiteau 1947 (incl. *Kitchingia* Baker): leaves and/or inflorescences of numerous taxa with bulbils.

More recently, the genus *Kalanchoe* has been recognized as consisting of three sections [5] or three subgenres [6]: *Bryophyllum*, *Kalanchoe* and *Kitchingia*.

*Kalanchoe* taxa recorded in Ecuador:

***Kalanchoe blossfeldiana*** Poelln., *Repert. Spec. Nov. Regni Veg*. 35: 159 (1934) ≡ *Kalanchoe globulifera* var. *coccinea* H. Perrier, *Arch. Bot. Bull. Mens*. 2(1): 26 (1928).

***Kalanchoe carnea*** N.E. Br., *Gard. Chron*., n.s. 1: 298 (1886).

***Kalanchoe crenata*** (Andrews) Haw., *Syn. Pl. Succ.*: 109. (1812) ≡ *Vereia crenata* Andrews, *Bot. Repos*. t. 21 (1798).

***Kalanchoe daigremontiana*** Raym.-Hamet & H. Perrier, *Ann. Mus. Colon. Marseille*, sér. 3, 2: 128–132 (1914) ≡ *Bryophyllum daigremontianum* (Raym.-Hamet & H. Perrier) A. Berger. *Nat. Pflanzenfam*. ed. 2, 18a: 412, fig. 197 (1930).

***Kalanchoe densiflora*** Rolfe, *Bull. Misc. Inform. Kew* 1919: 263 (1919) = *Kalanchoe bequaertii* De Wild., *Bull. Misc. Inform. Kew*: 263 (1919).

***Kalanchoe eriophylla*** Hils. & Bojer ex Tul., *Ann. Sci. Nat., Bot.*, sér. 4, 8: 149 (1857) = *Cotyledon pannosa* Baker, *J. Linn. Soc., Bot*. 18: 269 (1881).

***Kalanchoe fedtschenkoi*** Raym.-Hamet & H. Perrier, *Ann. Mus. Colon. Marseille*, sér. 3, 3: 75–80 (1915) ≡ *Bryophyllum fedtschenkoi* (Raym.-Hamet & H. Perrier) Lauz.-March., *Compt. Rend. Hebd. Séances Acad. Sci*. 278: 2508 (1974).

***Kalanchoe gastonis-bonnieri*** Raym.-Hamet & H. Perrier, *Ann. Sci. Nat., Bot.*, sér. 9, 16: 364 (1912) ≡ *Bryophyllum gastonis-bonnieri* (Raym.-Hamet & H. Perrier) Lauz.-March., *Compt. Rend. Hebd. Séances Acad. Sci*. 278: 2508 (1974) = *Kalanchoe adolphi-engleri* Raym.-Hamet, *Bull. Soc. Bot. France* 102: 239 (1955).

***Kalanchoe* ×*houghtonii*** D.B. Ward, *Cact. Succ.* *J.* 78(2): 94 (2006) ≡ *Bryophyllum houghtonii* (D.B. Ward) P.I. Forst, *Austrobaileya* 7(2): 383 (2006). Parentals: *Kalanchoe daigremontiana* (Lam.) Pers. and *Kalanchoe tubiflora* (Harv.) Raym.-Hamet

***Kalanchoe* ×*laetivirens*** Desc., *J. Bot. Soc. Bot. France* 4: 85 (1997), pro sp. ≡ *Bryophyllum laetivirens* (Desc.) V.V. Byalt, *Bot. Zhurn. (Moscow & Leningrad)* 93: 463 (2008). Parentals: *Kalanchoe daigremontiana* Raym.-Hamet & H. Perrier. (likely) and possibly *Kalanchoe laxiflora* Baker.

***Kalanchoe laxiflora*** Baker, *J. Linn. Soc., Bot*. 22: 472 (1887).

***Kalanchoe mortagei*** Raym.-Hamet & H. Perrier, *Bull. Soc. Bot. France* 95: 120 (1948) ≡ *Bryophyllum mortagei* (Raym.-Hamet & H. Perrier) Wickens, *Kew Bull*. 36(4): 672 (1981) ≡ *Kalanchoe poincarei* var. *mortagei* (Raym.-Hamet & H. Perrier) Boiteau, *Kalanchoe Madagascar*: 122 (1995).

***Kalanchoe pinnata*** (Lam.) Pers., *Syn. Pl*. 1: 446. (1805) ≡ *Cotyledon pinnata* Lam., *Encycl*. 2(1): 141 (1786) ≡ *Verea pinnata* (Lam.) Spreng., *Syst. Veg*., ed. 16, 2: 260 (1825) ≡ *Bryophyllum pinnatum* (Lam.) Oken, *Allg. Naturgesch*. 3(3): 1966 (1841) ≡ *Bryophyllum pinnatum* (Lam.) Kurz, *J. Asiat. Soc. Bengal, Pt. 2, Nat. Hist*. 40(2): 52 (1871) = *Bryophyllum calycinum* Salisb., *Parad. Lond*. pl. 3 (1805).

***Kalanchoe thyrsiflora*** Harv., *Fl. Cap*. t. II: 379 (1861).

***Kalanchoe tomentosa*** Baker, *J. Bot*. 20: 110 (1882).

***Kalanchoe tubiflora*** (Harv.) Raym.-Hamet, *Beih. Bot. Centralbl*. 29(2): 41 (1912) ≡ *Bryophyllum tubiflorum* Harv., *Fl. Cap*. 2: 380 (1862) = *Kalanchoe delagoensis* Eckl. & Zeyh. *Enum. Pl. Afr. Austral.* 3: 305 (1836).

**References**

1. Berger, P. Crassulaceae. Unterfam. II. Kalanchoideae [sic Kalanchooideae]. 6. Kalanchoe. In *Die natürlichen Pflanzenfamilien*, 2nd ed.; Engler, A., Prantl, K., Eds.; Verlag von Wilhelm Engelmann: Leipzig, Germany, 1935; pp. 402–412.
2. Fu, K.; Ohba, H. Crassulaceae. In *Flora of China*; Wu, Z.Y., Raven, P.H. Eds.; Science Press and Missouri Botanical Garden Press: Beijing, China, 2001; Volume 8, pp. 202–268.
3. Moran, R.V. Crassulaceae. In *Flora of North America North of Mexico*; Flora of North America Editorial Committee Ed.; Oxford University Press: New York, NY, USA, 2009; Volume 8, pp. 147–230.
4. Descoings, B. *Kalanchoe*. In *Illustrated Handbook of Succulent Plants: Crassulaceae*; Eggli, U., Ed.; Springer: Berlin, Germany, 2003; pp. 143–181.
5. Chernetskyy, M. Problems in Nomenclature and Systematics in the Subfamily Kalanchoideae (Crassulaceae) over the Years. *Acta Agrobot*. **2011**, *64*, 67–74.
6. Smith, G.F.; Figueiredo, E. The Infrageneric Classification and Nomenclature of Kalanchoe Adans. (Crassulaceae), with Special Reference to the Southern African Species. *Bradleya* **2018**, *36*, 162–172.
