# Supplementary figures and images for: The Genus Kalanchoe (Crassulaceae) in Ecuador: From Gardens to the Wild
Source: Plants (Basel). 2022 Jun 30;11(13):1746. doi: 10.3390/plants11131746 (PMC9269312; doi:10.3390/plants11131746)

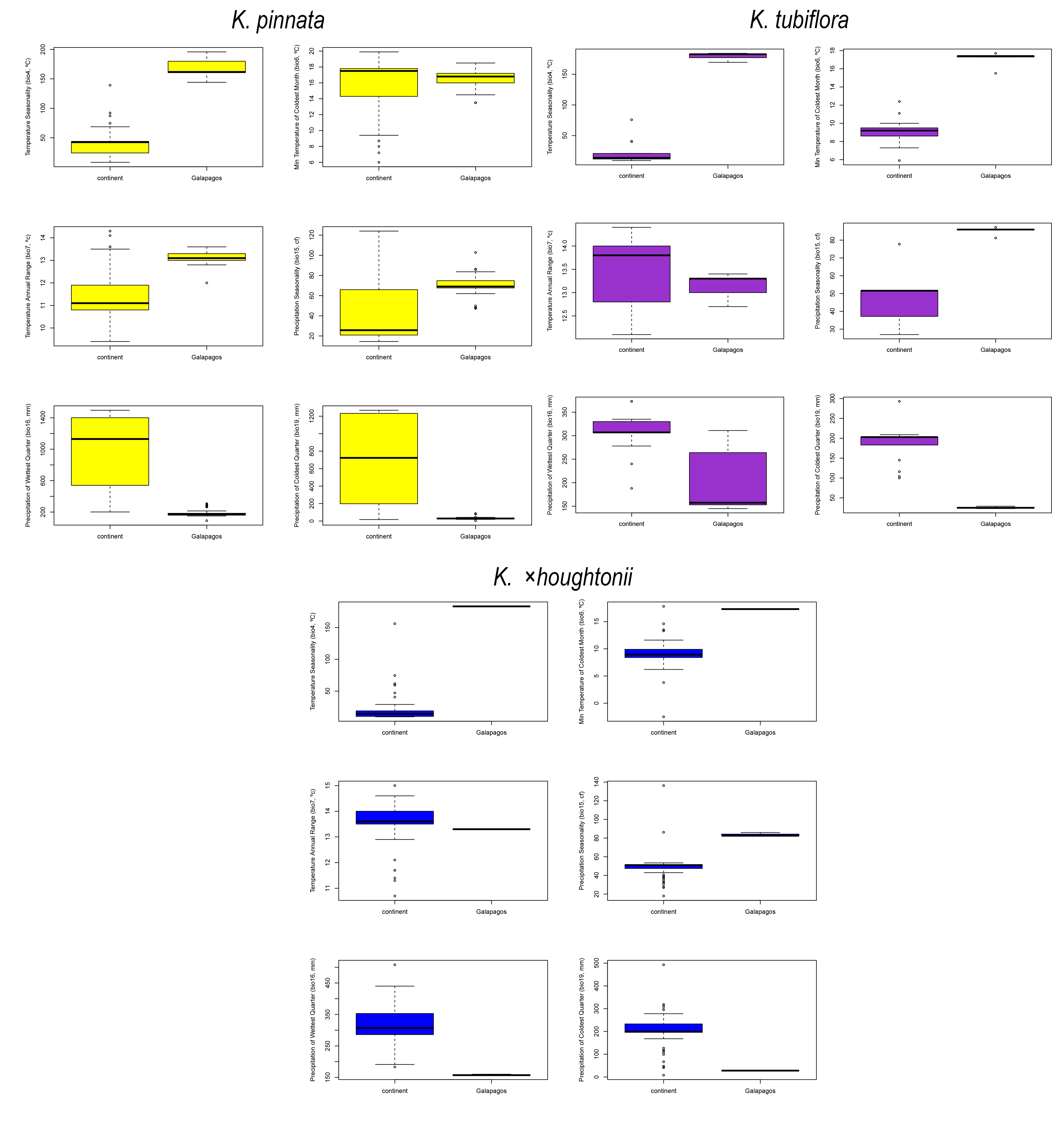

Supplement: Supplementary file 1 [file plants-11-01746-s001.zip › plants-1779913-supplementary/Figure S1.png]

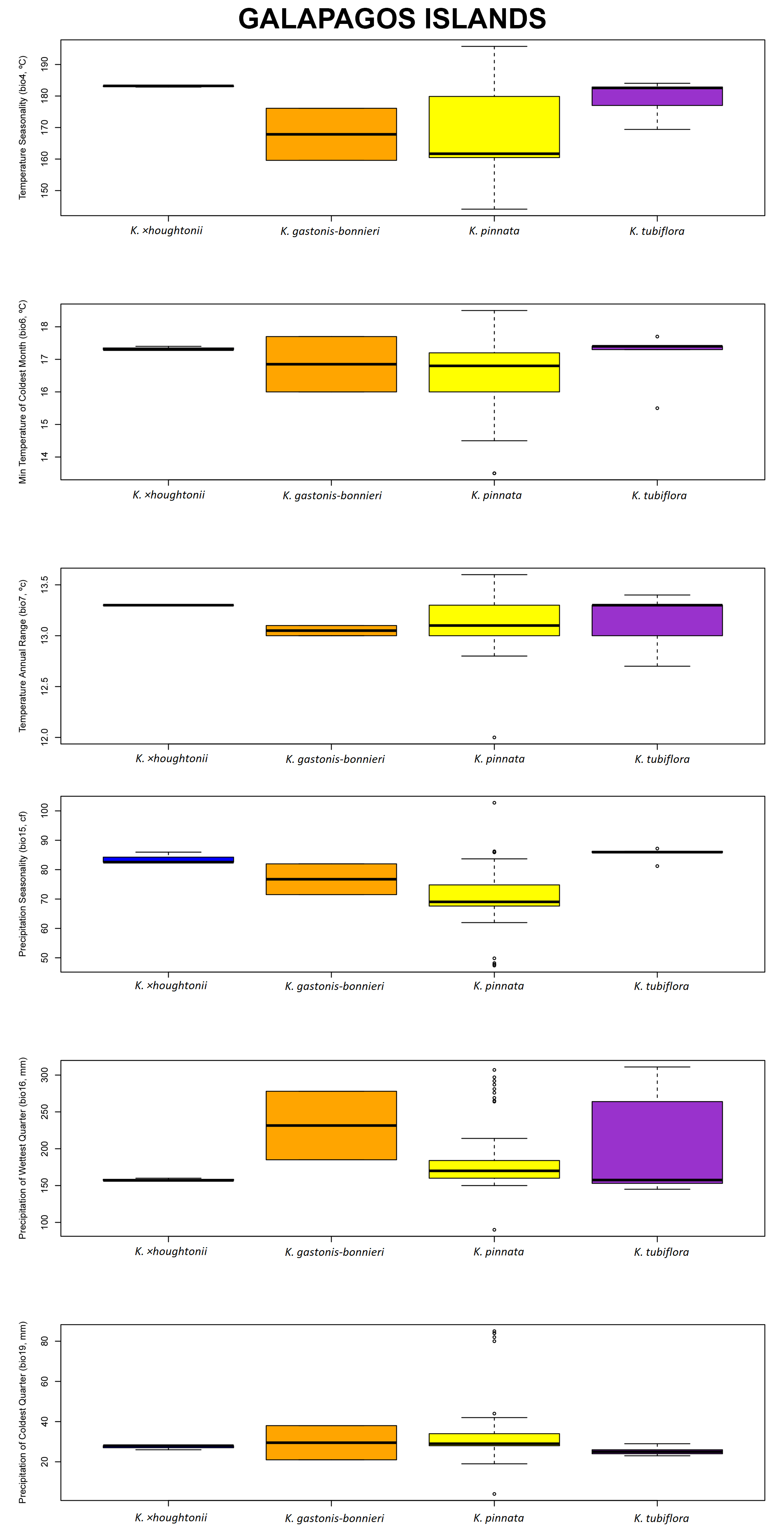

Supplement: Supplementary file 1 [file plants-11-01746-s001.zip › plants-1779913-supplementary/Figure S2.png]

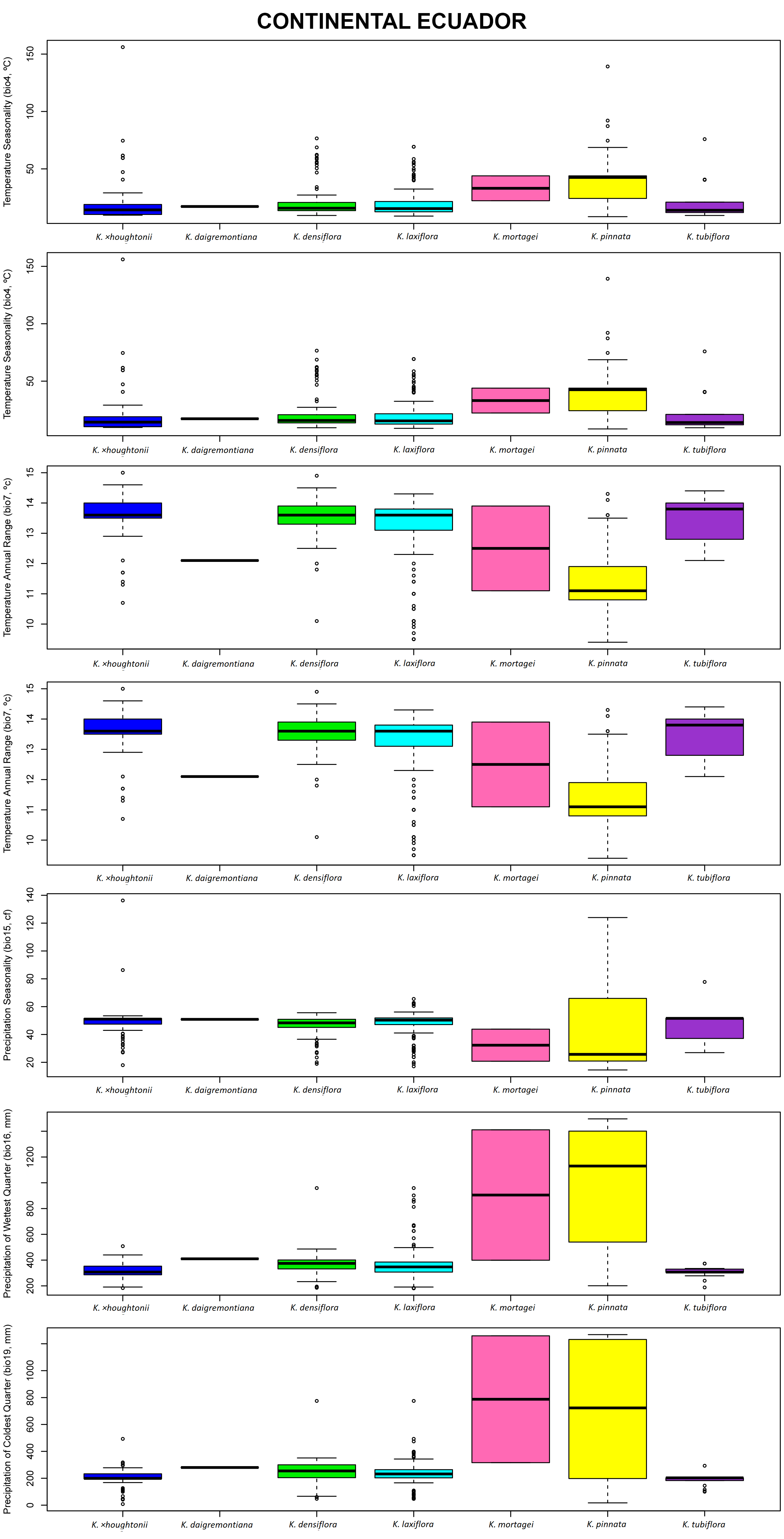

Supplement: Supplementary file 1 [file plants-11-01746-s001.zip › plants-1779913-supplementary/Figure S3.png]

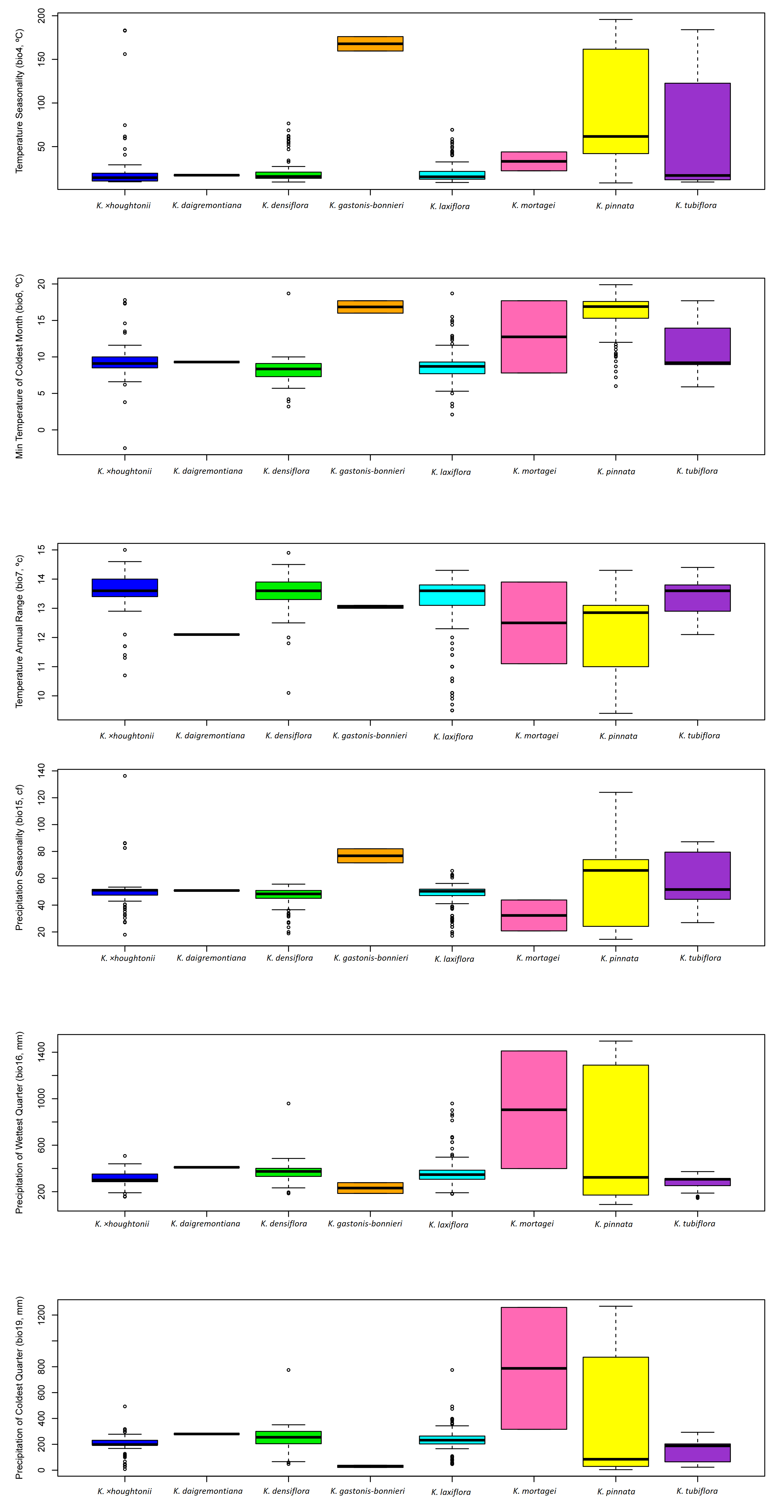

Supplement: Supplementary file 1 [file plants-11-01746-s001.zip › plants-1779913-supplementary/Figure S4.png]

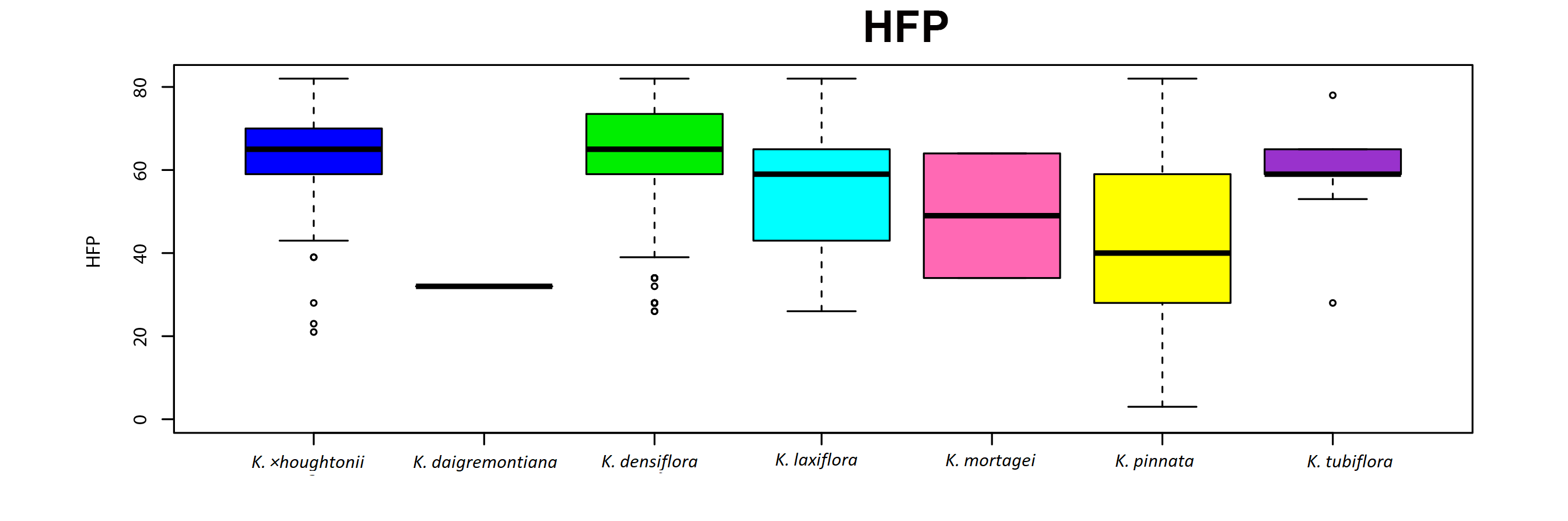

Supplement: Supplementary file 1 [file plants-11-01746-s001.zip › plants-1779913-supplementary/Figure S5.png]

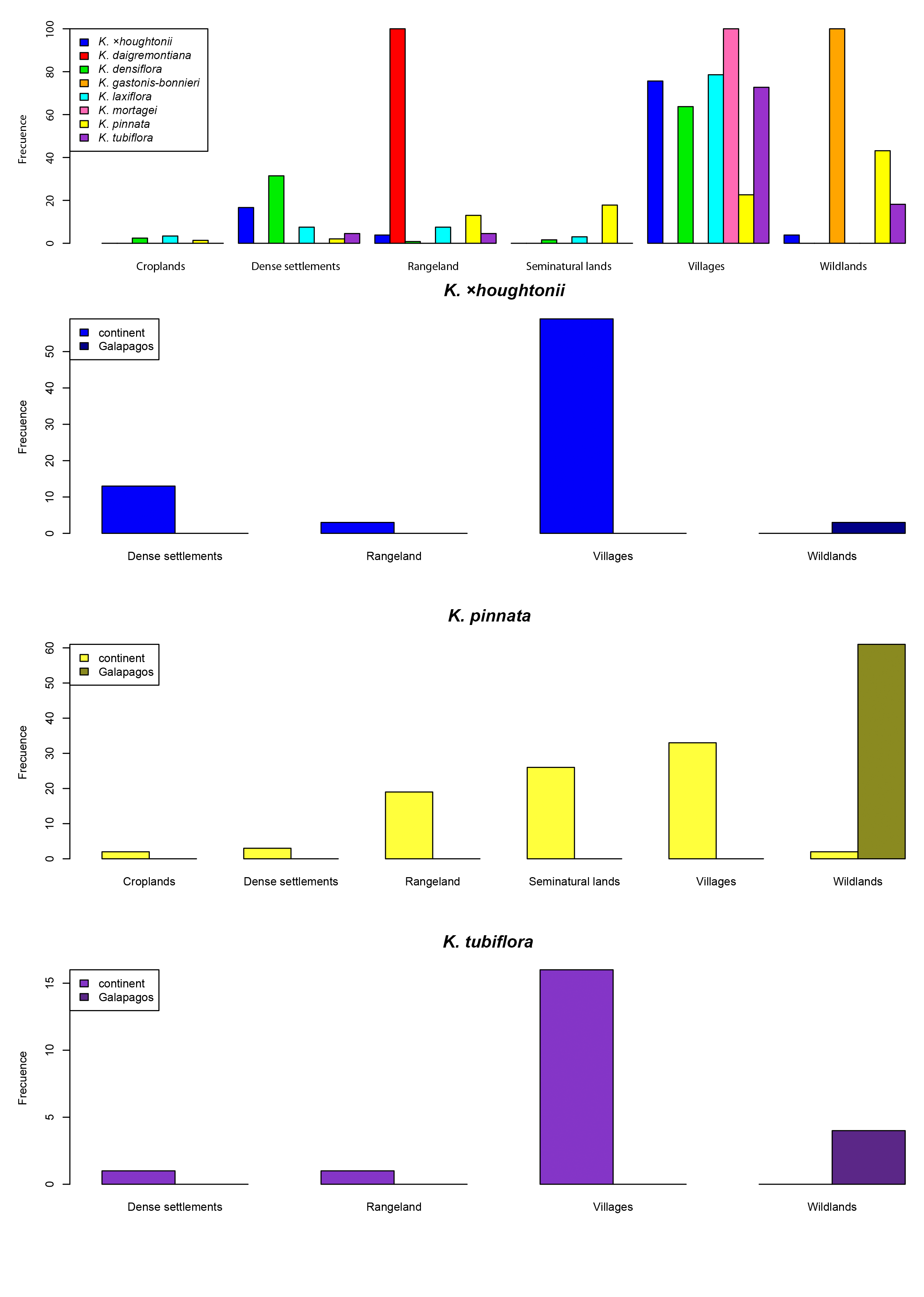

Supplement: Supplementary file 1 [file plants-11-01746-s001.zip › plants-1779913-supplementary/Figure S6.png]

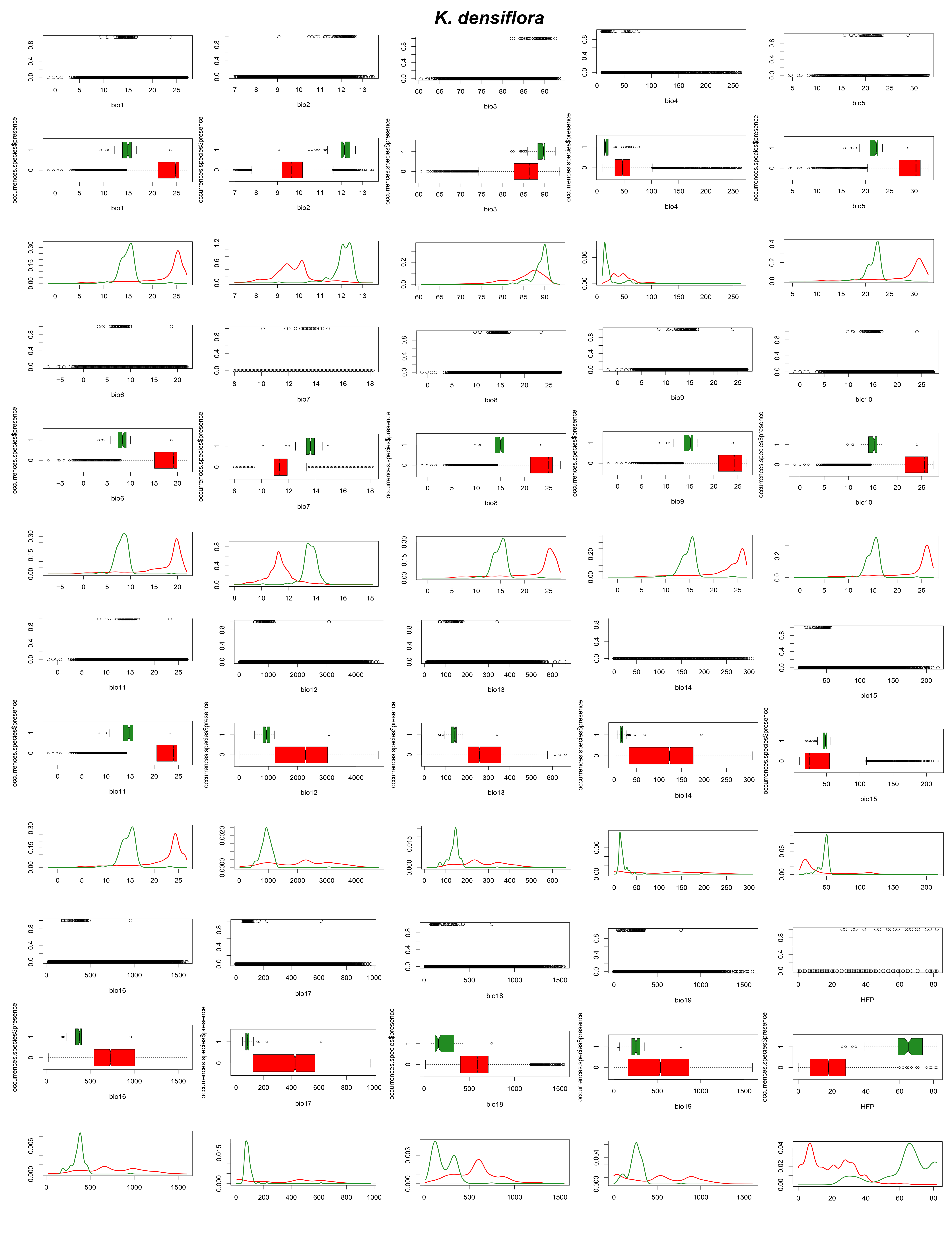

Supplement: Supplementary file 1 [file plants-11-01746-s001.zip › plants-1779913-supplementary/Figure S7 (1).png]

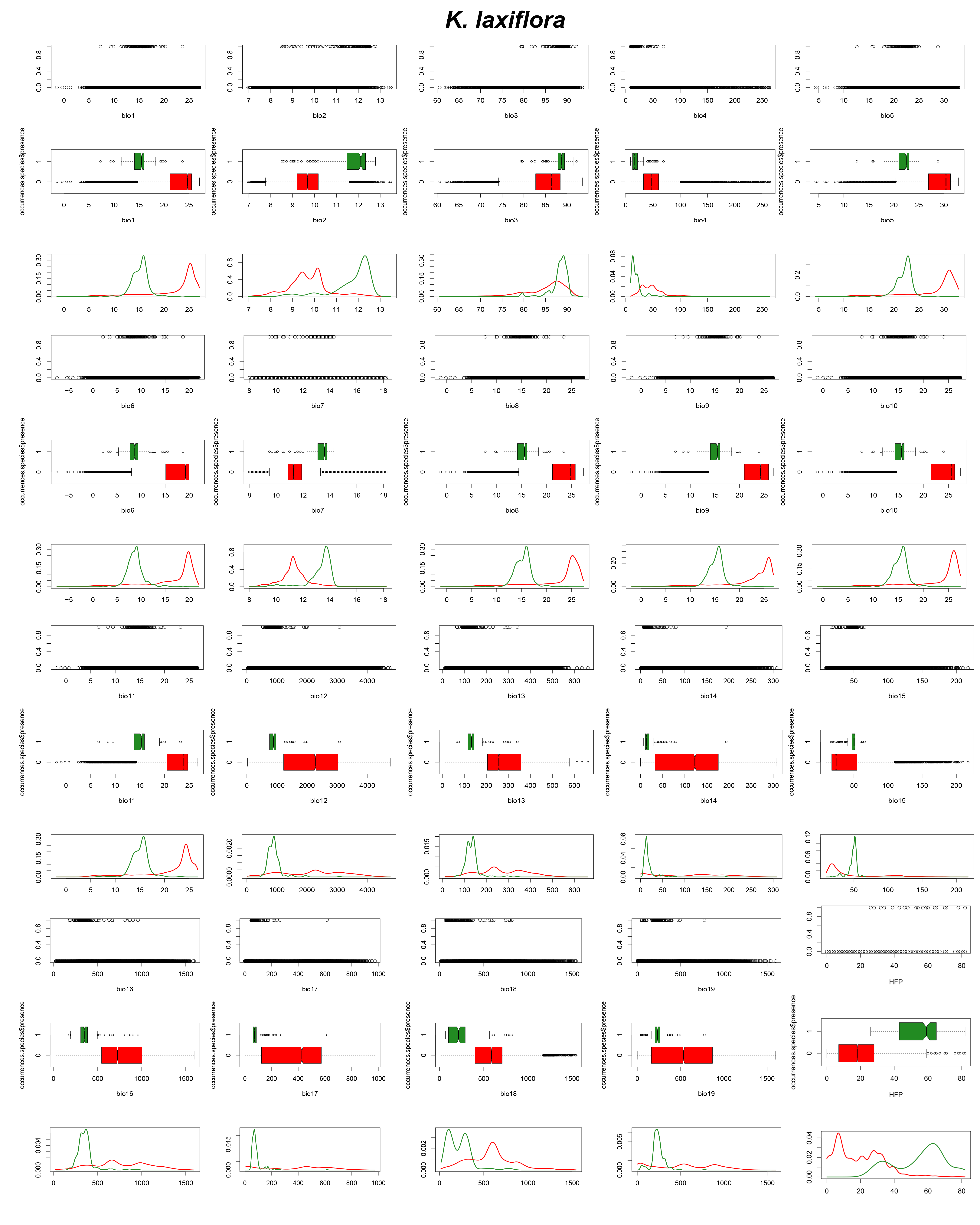

Supplement: Supplementary file 1 [file plants-11-01746-s001.zip › plants-1779913-supplementary/Figure S7 (2).png]

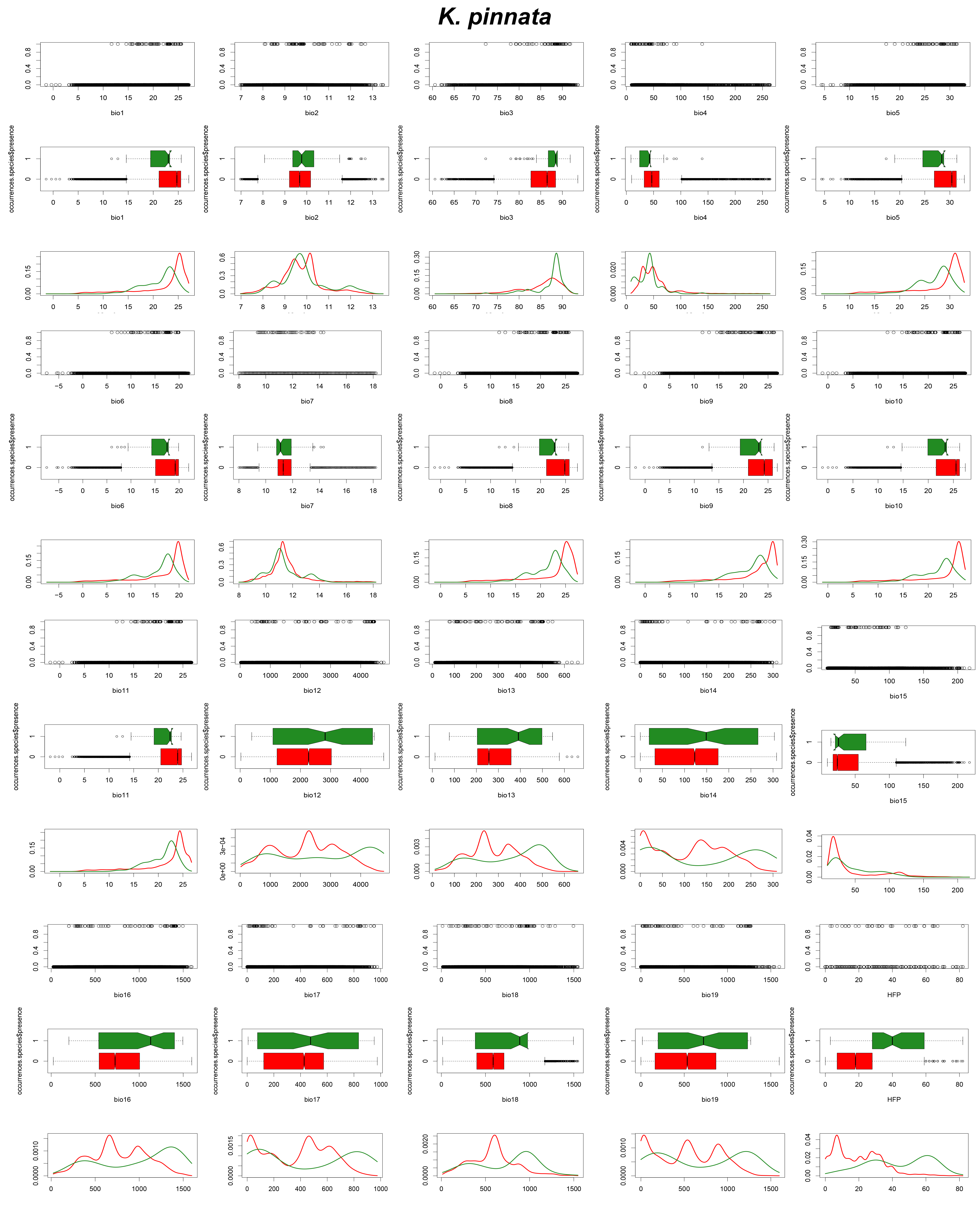

Supplement: Supplementary file 1 [file plants-11-01746-s001.zip › plants-1779913-supplementary/Figure S7 (3).png]

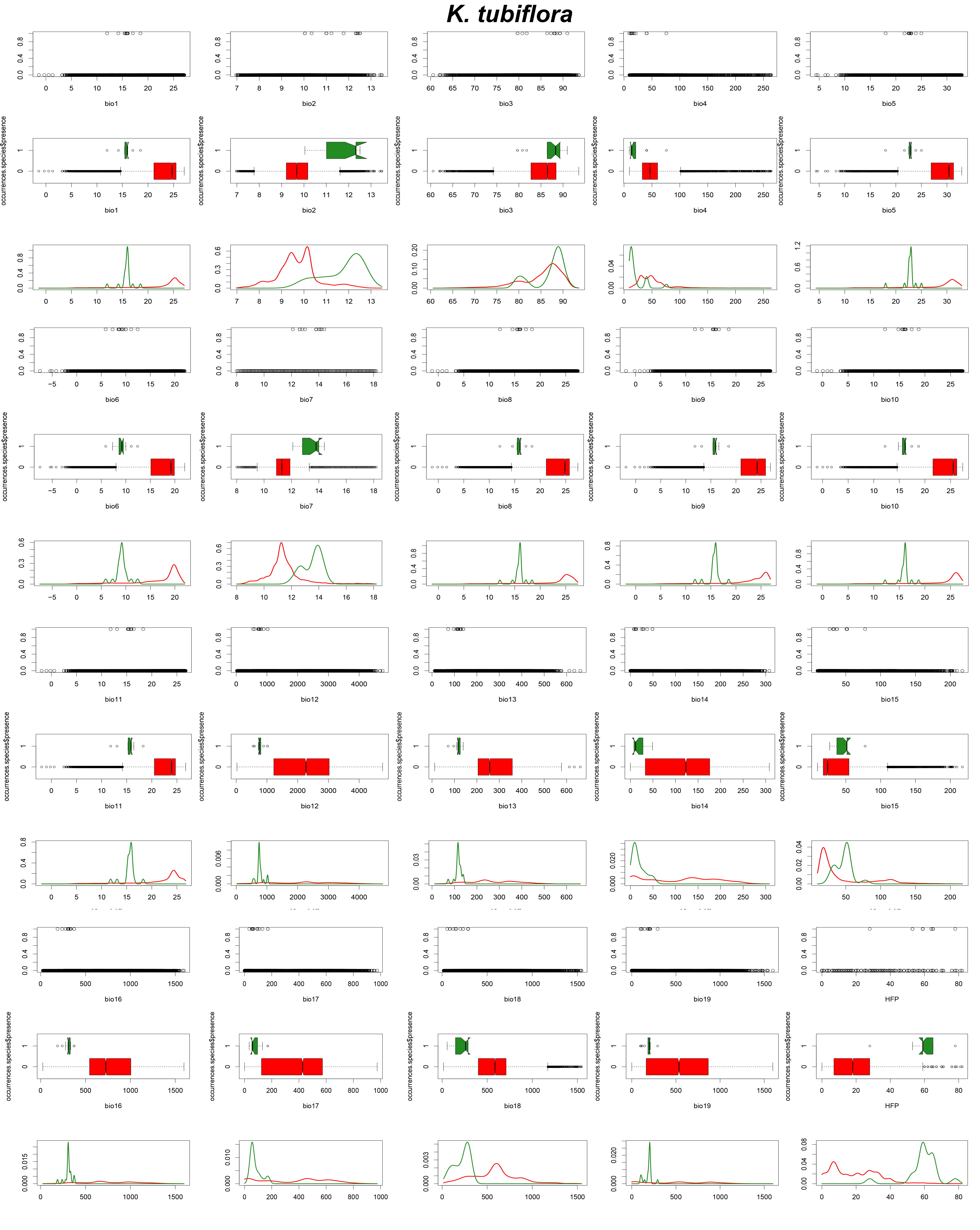

Supplement: Supplementary file 1 [file plants-11-01746-s001.zip › plants-1779913-supplementary/Figure S7 (4).png]

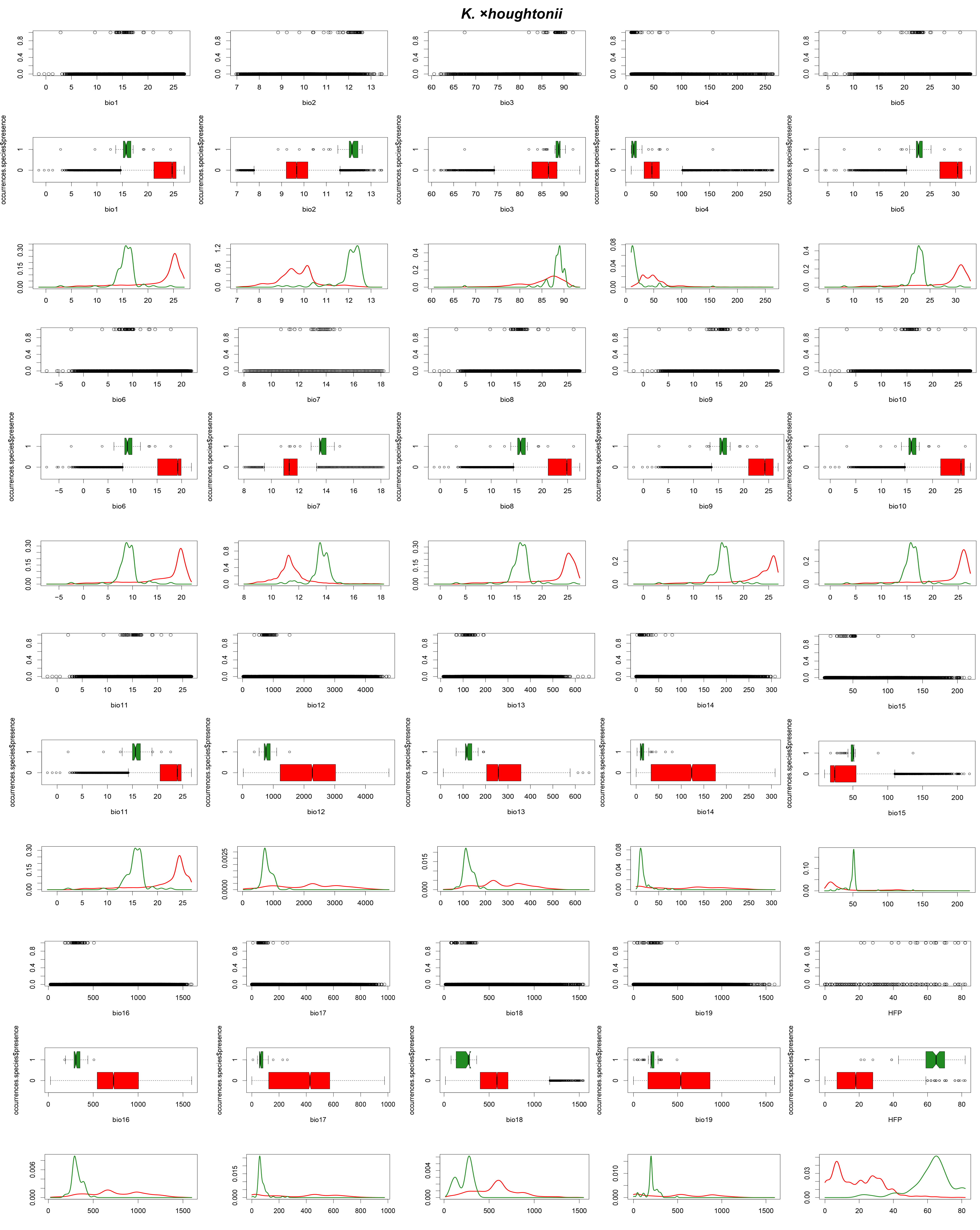

Supplement: Supplementary file 1 [file plants-11-01746-s001.zip › plants-1779913-supplementary/Figure S7 (5).png]
